# The PIFs Redundantly Control Plant Defense Response against *Botrytis cinerea* in *Arabidopsis*

**DOI:** 10.3390/plants9091246

**Published:** 2020-09-21

**Authors:** Shengyuan Xiang, Songguo Wu, Haiyan Zhang, Minghui Mou, Yanli Chen, Daibo Li, Houping Wang, Ligang Chen, Diqiu Yu

**Affiliations:** 1 CAS Key Laboratory of Tropical Plant Resources and Sustainable Use, Xishuangbanna Tropical Botanical Garden, Chinese Academy of Sciences, Menglun, Mengla 666303, China; xsy1174@163.com (S.X.); wusongguo@xtbg.ac.cn (S.W.); zhanghaiyan@xtbg.ac.cn (H.Z.); 13227495018@163.com (M.M.); chenyanli14787822@163.com (Y.C.); leedaibo@163.com (D.L.); hpwang234@163.com (H.W.); 2 University of Chinese Academy of Sciences, Beijing 100049, China; 3 Center of Economic Botany, Core Botanical Gardens, Chinese Academy of Sciences, Menglun, Mengla 666303, China

**Keywords:** *Arabidopsis*, *Botrytis cinerea*, defense response, PIF, ethylene, jasmonic acid

## Abstract

Endogenous and exogenous signals are perceived and integrated by plants to precisely control defense responses. As a crucial environmental cue, light reportedly plays vital roles in plant defenses against necrotrophic pathogens. Phytochrome-interacting factor (PIF) is one of the important transcription factors which plays essential roles in photoreceptor-mediated light response. In this study, we revealed that PIFs negatively regulate plant defenses against *Botrytis cinerea*. Gene expression analyses showed that the expression level of a subset of defense-response genes was higher in *pifq* (*pif1/3/4/5*) mutants than in the wild-type control, but was lower in *PIF*-overexpressing plants. Chromatin immunoprecipitation assays proved that PIF4/5 binds directly to the *ETHYLENE RESPONSE FACTOR1* (*ERF1*) promoter. Moreover, genetic analyses indicated that the overexpression of *ERF1* dramatically rescues the susceptibility of *PIF4-HA* and *PIF5-GFP* transgenic plants, and that PIF controls the resistance to *B. cinerea* in a COI1- and EIN2-dependent manner. Our results provide compelling evidence that PIF, together with the jasmonate/ethylene pathway, is important for plant resistance to *B. cinerea*.

## 1. Introduction

As sessile organisms, stress-resistant plants employ complex defense mechanisms to counter the adverse effects of multiple pathogens and/or herbivorous insects. The precisely controlled immunity is vital for the survivability of plants in the interaction with pathogens. According to the lifestyles, plant pathogens can be divided into biotrophs, which obtain nutrition from living host cells, and necrotrophs, which feed on the dead plant tissue [1]. As one of the typical broad host-range necrotrophic pathogens which can infect plants with no host specificity, *Botrytis cinerea* has been found to cause huge economical losses in agricultural production and is used as a model fungus to study the interaction between plants and pathogens. 

There has been considerable progress in characterizing the molecular mechanisms underlying immune signaling networks against *B. cinerea* over the past several years [2]. Specifically, the plant hormones jasmonate (JA) and ethylene (ET) play crucial roles in plant defenses against necrotrophic pathogens [1,2]. Jasmonate is a lipid-derived compound, perceived by the F-box protein CORONATINE INSENSITIVE1 (COI1), which forms the SCF^COI1^ E3 ubiquitin ligase with SKP1 and CULLIN1 to mediate the degradation of JAZ proteins [3,4,5,6]. JA-insensitive *coi1* mutant plants have compromised defense response against *B. cinerea* [7,8,9,10]. Activated JA signaling in plants is often associated with increased resistance against *B. cinerea*, as well as growth inhibition [11,12,13]. Moreover, JA has been showed to repress hypocotyl elongation in a COI1-, JAZ-, and MYCs-dependent manner [14]. Additionally, JAZs also contribute to the synergism between JA and ET signaling in response to necrotrophic pathogens by directly binding to EIN3/EIL1 [15], which are two major transcription factors affecting ET signaling. They function downstream of EIN2, which interacts with the ethylene receptor ETR1 to positively regulate ET signaling [16,17,18]. ET-insensitive *ein2* mutant plants are impaired in resistance to *B. cinerea* [7,8,19]. Following a *Botrytis cinerea* infection, JA and ET production are rapidly and simultaneously induced, leading to the activation of downstream defense-associated genes, such as *ERF1*, *OCTADECANOID-RESPONSIVE ARABIDOPSIS AP2/ERF-DOMAIN PROREIN 59* (*ORA59*), and *PLANT DEFENSIN 1.2* (*PDF1.2*), thereby enhancing plant disease resistance [11,12]. Moreover, simultaneous mutation of ethylene response factor ERF5 and ERF6 makes plants more susceptible against *B. cinerea* via compromised JA-induced defense gene expression [20].

In *A. thaliana*, a group of basic helix–loop–helix (bHLH) transcription factors called Phytochrome-interacting factor (PIFs) function downstream of phyB. The PIFs belong to subfamily 15 of the bHLH superfamily [21,22]. There are eight PIF proteins in Arabidopsis: PIF1, PIF2, PIF3, PIF4, PIF5, PIF6, PIF7, and PIF8 [23]. Recent functional analyses have provided some evidence that PIFs participate in a wide range of physiological processes, including seed germination, flowering, senescence, and shade avoidance [24,25,26,27,28,29,30,31,32]. As transcription factors, PIFs bind and regulate a large number of gene to regulate plant development and growth. For example, PIF negatively controls seed germination by decreasing gibberellin levels and increasing abscisic acid levels or by orchestrating ABA signaling in darkness [24,32,33]. Furthermore, PIF4 positively affects thermomorphogenesis and thermosensory-activated flowering [28,34], but negatively influences plant immunity [35]. Another study confirmed that PIF3/4/5 function as positive regulators during age-triggered and dark-induced leaf senescence [29]. Furthermore, PIF1/3/4/5 act redundantly to positively regulate the shade-avoidance syndrome by directly suppressing *MIR156* expression [30]. The *pifq* (*pif1/3/4/5*) plants show differential phenotypes when compared to wild-type in the process of growth, development, and resistance, including constitutive photomorphogenesis and less sensitivity to ABA-inhibited seed germination and primary root growth in darkness [32], attenuated shade-avoidance syndrome [30], and enhanced resistance to DC3000 [35].

Recently, some results have reported that the expression of *PIF3* and *PIF7* is suppressed during a *B. cinerea* infection [36], and that Phytochrome B integrates light hints into the jasmonate signaling pathway, to prioritize plant growth over defense, under competition environment [37], suggesting that PIFs have a role in regulating plant defense response. In this study, we demonstrated that PIFs redundantly control plant defenses against *B. cinerea* by modulating the expression of a subset of defense-response genes, among which *ERF1* can be directly bound and repressed by them. We also uncovered that the PIF-controlled defense is closely associated with JA/ET signaling.

## 2. Results

### 2.1. The Effect of PIF Mutations on Resistance to B. cinerea

**Figure 1:**
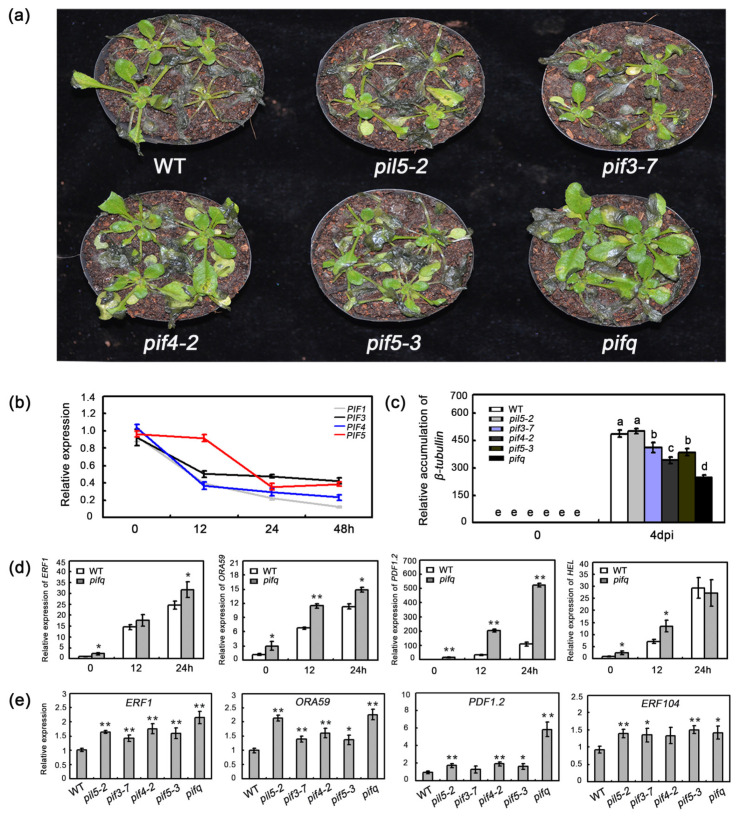
Phytochrome-interacting factor (PIF) proteins redundantly regulate plant defense response. (**a**) The disease phenotypes of *pif* mutant plants. The WT (Col-0) and *pif* plants were grown on soil for 28 days, under 12 h light/12 h dark conditions, and then inoculated with *B. cinerea* spores (5 × 10^5^ spores/mL); representative plants were photographed at six days post-inoculation (dpi). The experiments were repeated three times, with similar results. (**b**) The expression of *PIFs* was downregulated upon *Botrytis* inoculation. (**c**) The relative biomass of *Botrytis* in *pif* plants. RNA was isolated from spray-inoculated plants at 0 and 4 dpi, and the relative *β-tubulin* gene transcripts were examined. Error bars indicate SD of three independent experiments. The different letters above columns indicate significant differences (one-way ANOVA; *p* < 0.05). (**d**) The relative expression of *ERF1*, *ORA59*, *PDF1.2*, and *HEL* in WT and *pifq* after *Botrytis* inoculation. (**e**) The basal expression of *ERF1*, *ORA59*, *PDF1.2*, and *ERF104* in 14-day-old soil grown *pif* mutant under LD condition. For (**d**,**e**), error bars indicate SD of three independent experiments. Asterisks indicate Student’s *t*-test significant differences (* *p* < 0.05, ** *p* < 0.01).

The PIFs, which belong to the bHLH transcription factor family, negatively regulate phyB-mediated light signaling. Previous investigations that used a method of time series of transcriptomic analysis with high-resolution demonstrated that the expression of *PIF3* and *PIF7* is suppressed during *B. cinerea* infection [36]. To further investigate the function of PIFs in regulation of plant defense against *B. cinerea*, we first examined their expression, including *PIF1*, *PIF3*, *PIF4*, and *PIF5*, after treating plants with *B. cinerea*. As shown in Figure 1b, complementary to previous reports, we found that, in addition to *PIF3*, *B. cinerea* inoculation also greatly repressed the expression of *PIF1*, *PIF4*, and *PIF5*. To determine whether PIFs function in plant defenses against *B. cinerea*, we examined the WT plants, several single mutants (*pil5-2*, *pif3-7*, *pif4-2*, and *pif5-3*), and the *pifq* mutant. At six days post-inoculation, the symptom development of the *pil5-2*, *pif3-7*, and *pif5-3* plants was similar to that of the WT plants. In contrast, the *pif4-2* and *pifq* mutants had slightly and significantly less extensive disease symptoms, respectively, when compared with the WT plants (Figure 1a). Similarly, the *pif4-2* and *pifq* mutant plants had less pathogen biomass, when compared with the WT plants at three days post-inoculation (Figure 1c). Additionally, the *pifq* plants accumulated considerably less *β-tubulin* mRNA than the *pif* single mutants and the WT plants (Figure 1c). These results implied that PIFs function redundantly and negatively, to regulate plant defenses against *B. cinerea*.

### 2.2. The Effect of PIFs in Regulating the Basal and Pathogen-Induced Expression of Defense-Related Gene

**Figure 2:**
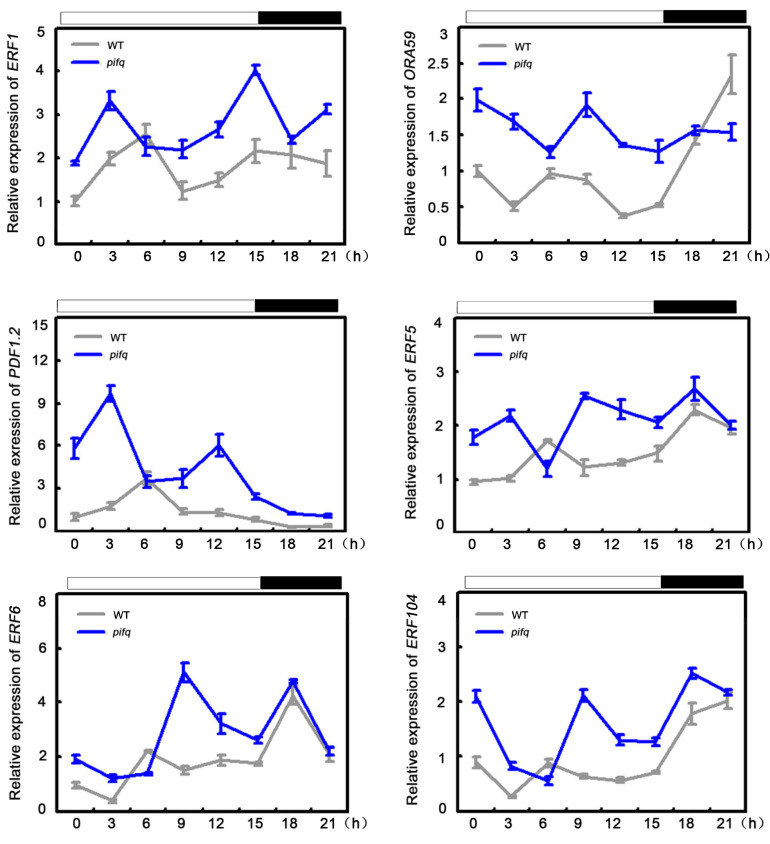
The basal expression of defense-response genes in WT and *pifq* plants. The plants were grown on soil, under LD condition, for 14 days. The plants for RNA isolation were harvested at given times from ZT0. *IPP2* gene was used as an internal control. Error bars indicate SD of three independent RNA extracts.

To defend against necrotrophic pathogens, plants activate a set of defense-related genes, including *ERF1, ORA59*, *PATHOGEN INDUCIBLE PLANT DEFENSIN* (*PDF1.2*), and *HEVEIN-LIKE PROTEIN* (*HEL*) [11,12,38]. To explore the molecular basis of the altered responses of the *pifq* mutants to the necrotrophic fungal pathogen, we first analyzed the expression of several defense-related genes in these plants, after an infection by *B. cinerea*. The *ERF1*, *ORA59*, *PDF1.2*, and *HEL* expression levels were higher in the *pifq* plants than in the WT plants, following the *B. cinerea* infection (Figure 1d). As shown in Figure 1d, it is obvious that *pifq* showed significantly high basal expression of these four genes. Thus, we hypothesized that *pif* mutants might have a more powerful basal defense. To further investigate this possibility, we tested the expression of defense-related genes in *pif* plants. The *pif* mutants we used showed a high expression level of defense-response genes, including *ERF1*, *ORA59*, *PDF1.2*, and *ERF104* (Figure 1e). Moreover, previous studies proved that the expression levels and the protein abundance of *PIFs* are affected by diurnal conditions [39,40,41,42]. Because of their functional redundancy, we then used *pifq* mutants to examine the expression levels of several defense-related genes during a 24 h period. The expression levels of defense-associated genes, such as *ERF1*, *ORA59*, *PDF1.2*, *ERF5*, and *ERF6*, were higher in the *pifq* plants than in the WT plants at the ZT0~ZT3 and ZT9~ZT12 time-points (Figure 2). Considering the clearly close correlation between high expression of defense response genes under both conditions with and without *B. cinerea* treatment and the largely resistant phenotypes after its infection in *pifq* plants, we concluded that PIFs regulate defense-associated genes expression to control plant resistance against *B. cinerea*.

### 2.3. The Effect of PIF Overexpressions on Resistance to B. cinerea

**Figure 3:**
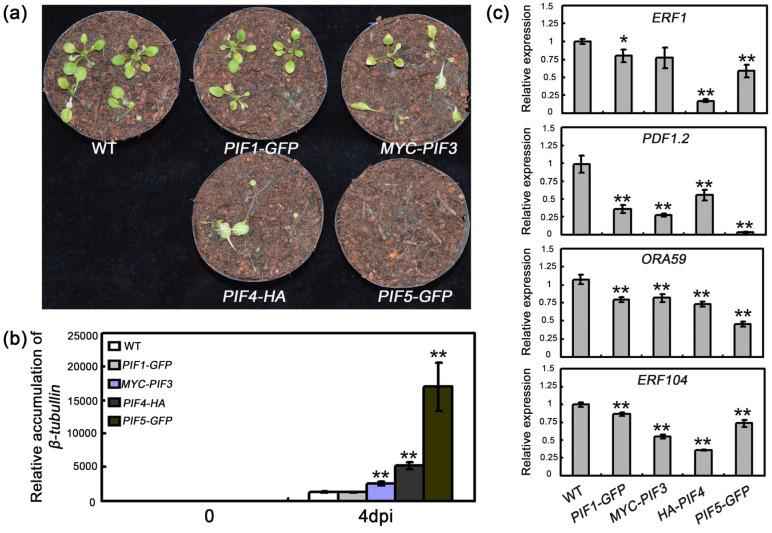
The *PIF* overexpression plants were more susceptible against *Botrytis* than WT. (**a**) The disease symptoms of *PIF* overexpression plants. The plants were grown under 12 h light/12 h dark conditions, on soil, for 14 days, and then inoculated with 1 × 10^5^ spores/mL *B. cinerea* spores. (**b**) The relative biomass of *Botrytis* in *PIF* overexpression plants. Error bars indicate SD of three independent experiments. (**c**) The basal expression of *ERF1*, *ORA59*, *PDF1.2,* and *ERF104* was analyzed in 14-day-old soil grown PIF overexpression plants under LD condition. Error bars indicate SD of three independent experiments. For (**b**,**c**), asterisks indicate Student’s *t*-test significant differences (* *p* < 0.05, ** *p* < 0.01).

To further characterize the role of PIFs in defense responses to *B. cinerea*, we also examined the basal expression of defense-associated genes in transgenic plants overexpressing *PIF1-GFP*, *MYC-PIF3*, *PIF4-HA*, or *PIF5-GFP*. As shown in Figure 3c, these overexpression plants had lower expression of *ERF1*, *ORA59*, *PDF1.2*, and *ERF104*, as compared to WT. Then we compared the pathogen growth in these transgenic plants with that in WT plants. At four days after an inoculation with *B. cinerea*, we observed a rapid increase in the necrotic symptoms and *β-tubulin* mRNA accumulation in the plants overexpressing *MYC-PIF3*, *PIF4-HA*, or *PIF5-GFP* (Figure 3a,b). Thus, the constitutive overexpression of *PIF* genes decreased the resistance of transgenic plants to *B. cinerea* and accelerated disease symptom development, suggesting that PIFs play an important role in plant defenses against *B. cinerea*.

### 2.4. Expression Profiling to Identify the Potential PIF-Involved Pathway to Control Defense Response

**Figure 4:**
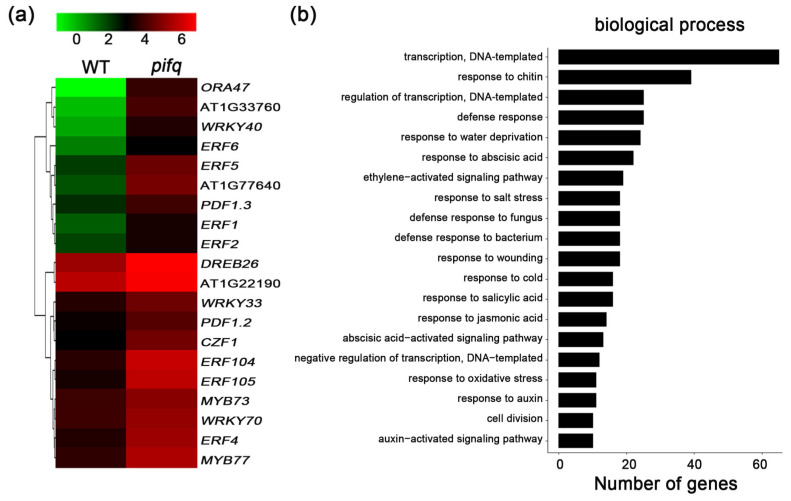
The PIFs-regulated genes involved in defense response. (**a**) The hierarchical cluster analysis of select upregulated transcripts that are involved in jasmonate/ethylene (JA/ET)-related defense or response to chitin/fungus. (**b**) Gene ontology analysis of the upregulated genes in *pifq*.

The obvious differences in the symptom development of the *pifq* mutant and *PIF*-overexpressing plants compelled us to further investigate the molecular basis of the altered responses. The basal expression levels of the defense-associated genes were higher and lower in the *pifq* and *PIF-*overexpressing plants, respectively (Figure 1e and Figure 3c). Thus, we completed a genome-wide transcriptomic analysis to compare the gene expression profiles between WT and *pifq* plants. The analysis revealed 409 differentially expressed genes of the *pifq* plants with more than two-fold changes in expression, including 137 downregulated genes and 272 upregulated genes (Supplementary Materials Data S1). Consistent with the enhanced disease resistance of the *pifq* plants, JA- and ET-related defense genes were among the 272 upregulated genes, including *ERF1*, *ERF5*, *ERF6*, *WRKY33*, *PDF1.2*, and *PDF1.3* (Figure 4a; Supplementary Materials Data S1). A gene ontology (GO) analysis functionally annotated genes with representative GO terms associated with defense responses to fungi or JA and the ethylene-activated signaling pathway (Figure 4b), suggesting that PIFs might regulate plant defenses against *B. cinerea* by modulating the transcription of defense-associated genes and that the function of PIFs to regulate defense response might be highly involved in the JA and ET signaling pathway.

### 2.5. In Vivo Interaction between PIF4/5 and the ERF1 Promoter

**Figure 5:**
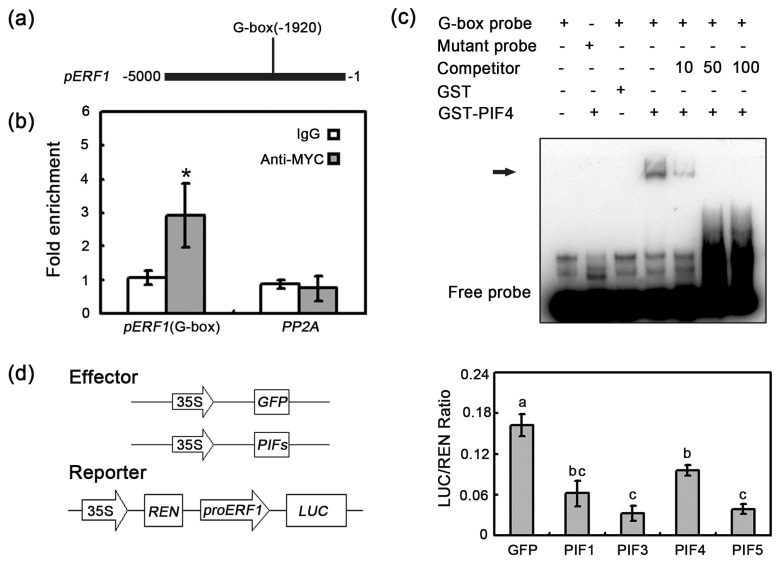
PIF4 directly binds to *ERF1* promoter and suppresses its expression. (**a**) Schematic diagram of the *ERF1* promoter. The solid line indicates the position of G-box in the promoter. (**b**) ChIP-qPCR, using *pPIF4-PIF4-MYC/pifq* plants, shows that PIF4 binds to the *ERF1* promoter region containing the G-box in vivo. The error bars indicate the SD of three independent experiments. Asterisks indicate Student’s *t*-test significant differences (* *p* < 0.05). (**c**) EMSA assay shows that GST-PIF4 bHLH recombinant protein binds to the promoter of *ERF1* in vitro. DNA fragments of *ERF1* promoter were synthesized with normal G-box motif (G-box probe) and mutated G-box motif (mutant probe), and labeled with biotin. The GST protein was used as negative control. (**d**) The diagram of the reporter and effectors used in the protoplast transfection assays. Transient expression assays indicate that PIFs repress *ERF1* expression. The error bars indicate SD of three independent experiments. The different letters above columns indicate significant differences (one-way ANOVA; *p* < 0.05).

Previous reports indicated that PIFs function by binding directly to the G-box (CACGTG) of their target gene promoters [30,34,43,44,45]. Given the existence of a G-box in the *ERF1* promoter (Figure 5a) and the negative regulation of *ERF1* expression by PIFs, we assumed that PIFs suppress *ERF1* expression by binding directly to the promoter. To confirm this assumption, we first conducted chromatin immunoprecipitation (ChIP) assays with transgenic lines expressing *pPIF4-PIF4-MYC* in *pifq* background and *PIF5-HA* under the control of the cauliflower mosaic virus (CaMV) 35S promoter. The ChIP-qPCR results further demonstrated that PIF4 and PIF5 can bind to the *ERF1* promoter via the G-box (Figure 5b and Supplementary Materials Figure S1a). Furthermore, to examine whether *ERF1* is directly targeted by PIF4 and PIF5 in vitro, gel electrophoresis mobility shift assay (EMSA) was performed. The GST-PIF4/5 specifically bound to the *ERF1* promoter region containing the normal G-box sequence (Figure 5c and Supplementary Materials Figure S1b). Additionally, with increasing concentrations, the unlabeled competitor greatly inhibited the binding of GST-PIF4/5. These observations indicated that PIFs can bind directly to the *ERF1* promoter.

To confirm the negative regulatory functions of PIFs, we performed transient expression assays in WT *A. thaliana* mesophyll protoplasts, using a dual-luciferase reporter plasmid. As a reporter, the *ERF1* promoter was fused to the firefly luciferase (*LUC*) gene, whereas the Renilla luciferase (*REN*) gene was placed under the control of the CaMV 35S promoter. The effector constructs contained *PIF* genes or *GFP* driven by the constitutive CaMV 35S promoter. The coexpression of *PIF* genes with the *proERF1-LUC* reporter significantly decreased the LUC/REN ratio relative to the effects of *GFP* (Figure 5d). This result further supported that PIFs act as negative regulators to repress the expression of *ERF1* and suggested that there is a possibility that PIFs control plant defense response through directly inhibiting the expression of other defense-related genes.

### 2.6. Association of PIF-Regulated Defense with JA/ET Signaling

**Figure 6:**
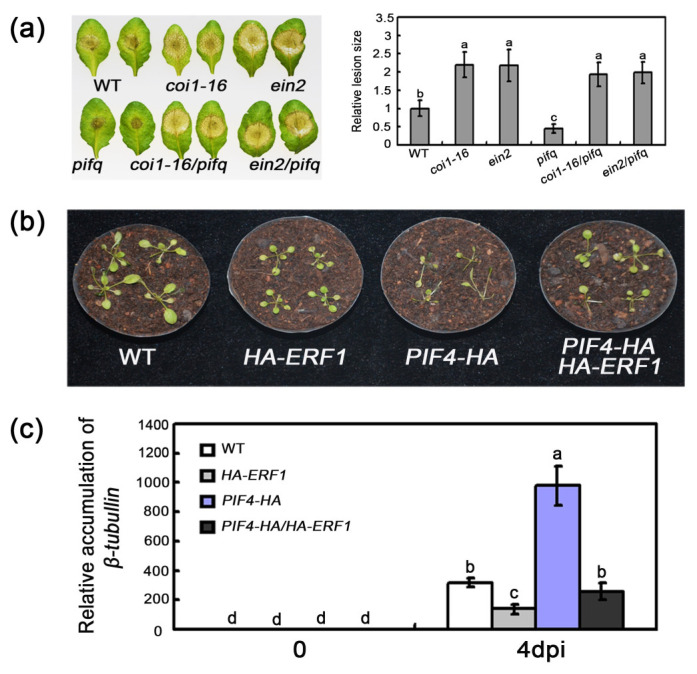
The functional relationship between PIFs and JA/ET in defense response. (**a**) The disease symptoms of drop-inoculated detached leaves were taken at 3 dpi, and the relative lesion size of pathogen on leaves were also measured. Error bars indicate SD of more than 15 leaves. (**b**) *HA-ERF1* can partially rescue the enhanced susceptibility of *PIF4-HA* plants against *Botrytis*. (**c**) The *B. cinerea β-tubulin* mRNA accumulation analysis. Error bars indicate SD of three independent experiments. For (**a**,**c**), the different letters above columns indicate significant differences (one-way ANOVA; *p* < 0.05).

Having ascertained that PIFs regulate the expression of several JA/ET-related defense genes following an infection by *B. cinerea* and the GO terms regarding JA/ET response enriched in transcriptome analysis, we examined whether the enhanced defense response of *pifq* plants is also associated with JA/ET signaling pathways. Specifically, *coi1-16* and *ein2* mutants were crossed with *pifq* plants, to generate *coi1-16/pifq* and *ein2/pifq* quintuple mutants. The defense responses of these quintuple mutants were similar to those of the *coi1-16* and *ein2* plants, with larger lesions compared with the *pifq* plants (Figure 6a), indicating that PIF-regulated defense response against *B. cinerea* relies on JA and ET signaling. Additionally, we explored the genetic relationship between *PIF4/5* and *ERF1*. Plants overexpressing *PIF4-HA* and *PIF5-GFP* were crossed with a transgenic plant carrying the *HA-ERF1* construct. The overexpression of *ERF1* significantly decreased the disease susceptibility and pathogen biomass of the *PIF4-HA* and *PIF5-GFP* transgenic plants (Figure 6b,c and Supplementary Materials Figure S2). Thus, our results provide evidence that the PIF-mediated defense is dependent on JA and ET signaling pathway.

## 3. Discussion

Previous studies proved that phyB negatively regulates PIF transcription factors, at the protein level, by enhancing their degradation and by sequestering them from their target promoters [46]. Thus, loss-of-function *phyB* mutants show exaggerated PIF-mediated growth [47,48,49]. Another study also revealed that *phyb* mutants exhibit downregulated expression of JA-inducible genes, such as *HEL*, *ERF1*, and *PDF1.2*, and compromised resistance to *B. cinerea* [49,50,51]. Additionally, PIF4 was recently reported to positively regulate the temperature-induced suppression of defense responses to *Pto* DC3000 [35]. Therefore, deciphering the possible roles of PIFs in defense responses to necrotrophic pathogens will provide new insights into our understanding of the phyB-PIF module-mediated plant defenses.

Pathogen attack leads to extensive transcriptional changes and the production of specialized metabolites contributing to the establishment of effective plant defenses. Transcription factors critically influence plant innate immunity. Notably, the ethylene response factor transcription factors, such as ERF1 and ORA59, are key integrators of JA and ET defense signaling pathways [52]. In the current study, the expression levels of several *ERF* genes, including *ERF1* and *ORA59*, were downregulated in *35S:PIF* transgenic plants and upregulated in *pifq* mutants. This expression model is consistent with the disease resistance of these lines (Figure 1, Figure 2 and Figure 3), suggesting that PIFs negatively regulate *ERF* gene expression. Furthermore, a genetic analysis demonstrated that *35S:ERF1* can dramatically enhance disease resistance of *PIF4-HA* and *PIF5-GFP* transgenic plants (Figure 6b,c and Supplementary Materials Figure S2). All of these results, together, suggest that *PIF*s act upstream of *ERF1* to negatively regulate the resistance to this necrotrophic pathogen. Our transcriptome sequencing analysis also indicated that the expression levels of the defense-response genes are widely upregulated. The GO functional annotations revealed that categories associated with JA/ET signaling are enriched among the 409 differentially expressed genes in *pifq* plants, implying the PIF-mediated defense against *B. cinerea* is closely related to JA/ET signaling (Figure 4). Similarly, mutations to *COI1* and *EIN2* in *pifq* plants dramatically compromised the resistance to *B. cinerea* (Figure 6a), further indicating that PIFs may be incorporated in the JA/ET pathway to control plant resistance to *B. cinerea*.

Numerous studies have demonstrated that the PIF proteins perform their biological functions by directly binding to the G-box (CACGTG) in their target promoters [30,34,43,44,45]. The *ERF1* promoter contains a G-box *cis*-elements. Our EMSA and ChIP experiments revealed that PIF4/5 can bind directly to the G-box in the *ERF1* promoter (Figure 5b,c and Supplementary Materials Figure S1), suggesting that *ERF1* is a direct target of PIF. The opposite expression patterns of *ERF1* in *PIF* mutants and overexpressing lines, as well as the downregulation of *LUC* expression in the transient expression assays (Figure 1e, Figure 3c and Figure 5d), further suggest that PIFs are negative regulators of *ERF1* expression. Thus, our results provide evidence that PIFs may function as negative regulators of plant defenses against *B. cinerea* via the direct inhibition of *ERF1* expression. To more thoroughly characterize the biological functions of PIFs and their possible signaling pathways in defense responses to *B. cinerea*, their downstream target genes will need to be identified. Moreover, the 409 differentially expressed genes in *pifq* plants may include other PIF targets. Previous studies indicated that PIF proteins can function as both positive and negative regulators [24,28,29,30,32,33,34,35]. Thus, PIFs extensively participate in the fine-tuning and tight control of the complex signaling and transcriptional networks that mediate plant growth and stress responses by functioning as positive and negative regulators.

## 4. Materials and Methods

### 4.1. Plant Materials and Growth Conditions

*Arabidopsis thaliana* ecotype Columbia-0 was used in this study. The T-DNA insertion mutants *pif1* (SALK_131872C), *pif3-7* (CS66042), *pif4-2* (CS66043), *pif5-3* (CS66044), and *pif1/3/4/5* (CS66049) were purchased from Arabidopsis Biological Resource Center. The overexpression transgenic plants involved in this study have been described: *MYC-PIF3* [53], *PIF4-HA*, *PIF5-HA* [30], *HA-ERF1* [54], *coi1-16*, and *ein2*. To generate *PIF1-GFP* and *PIF5-GFP* overexpression plants, Col-0 was transformed with p*35S:PIF1*-GFP and p*35S:PIF5*-GFP constructs. The *coi1-16/pif1/3/4/5*, *ein2/pif1/3/4/5*, *phyb/pif1/3/4/5*, *PIF4-HA/HA-ERF1*, and *PIF5-GFP/HA-ERF1* were prepared by genetic crossing. The *Arabidopsis* seeds were sterilized in 10% bleach and sown on 1/2 MS medium with 0.5% agar and 1% sucrose (pH 5.8). After stratification for 3 days, at 4 °C, seeds were then transferred to growth chamber and grown under 16 h light/8 h dark (LDs) or 12 h light/12 h dark at 22 °C. Primers designed for mutant identification or clones are listed in Supplementary Materials Table S1.

### 4.2. Pathogen Infection

*Botrytis cinerea* (B05.10) was grown on Potato Dextrose Agar, under 12 h light/12 h dark, at 21 °C. *B. cinerea* spores’ collection and inoculation of plants were performed as previously described [10]. The inoculated plants were maintained in a dark and high-humidity environment. After 3 to 7 days, the symptom development could be observed. *B. cinerea* growth was quantified by qRT-PCR of total RNA isolated from the inoculated plants. For drop inoculation, leaves of 28-day-old plants grown on soil were inoculated with a single 8 μL drop of a suspension of 5 × 10^5^ spores/mL, in Sabouraud maltose broth (SMB) buffer. The lesion sizes of *B. cinerea* infected leaves were measured, using ImageJ.

### 4.3. Expression Analysis

For reverse-transcription PCR analysis, total RNA was extracted from *Arabidopsis* seedlings, using the TRIzol reagent (Invitrogen, Waltham, MA, USA). Then, cDNA was synthesized from 1 μg of total RNA, according to the reverse-transcription protocol (Takara, Beijing, China). The cDNA was subjected to qPCR, using the SYBR Premix Ex Taq (Takara, Beijing, China), on a Roche LightCycler 480. *ACTIN2* or *IPP2* was amplified as the reference gene. The 2^−ΔΔCt^ method was used for the calculation of relative expression levels. At least three biological replicates were conducted for each experiment. The gene-specific primers are provided in Supplementary Materials Table S1.

### 4.4. RNA Sequencing

The rosettes of 14-day-old WT and *pifq* plants grown on soil were collected. The total RNA was extracted from *Arabidopsis* seedlings, using the Trizol reagent (Invitrogen, Waltham, MA, USA). Shanghai OE Biotech Co. provided the supports of RNA sequencing and data analysis. TruSeq Stranded mRNA LTSample Prep Kit (Illumina, San Diego, CA, USA) was used to construct the libraries. Then the sequencing was performed, using Illumina HiSeqTM 2500. Trimmomatic was used to filter the raw sequencing reads. The differential genes were analyzed by DESeq, with *p* < 0.05 and fold change > 2 as the threshold. The GOseq was used to perform GO enrichment analysis of the differential genes. The hierarchical cluster of selected genes was constructed by using R package pheatmap.

### 4.5. Gel Mobility Shift Assay

For EMSA assay, PIF4 bHLH domain and the full-length PIF5 coding sequences were cloned into pGEX-TX-1 vector and transformed into *E. coli* strain transetta (DE3) (TransGen, Beijing, China). The recombinant proteins were induced at 37 °C for 3 h, with 0.1 mM IPTG, and purified with Glutathione-agarose beads (TransGen, Beijing, China). The 5′ terminal biotin-labeled DNA fragments were synthesized. The EMSA assay was performed, using Chemiluminescent EMSA Kit (Beyotime, Shanghai, China), following the manufacturer’s protocol.

### 4.6. Chromatin Immunoprecipitation Assay

For ChIP analyses, 18-day-old soil-grown plants, under LD conditions, were harvested at ZT0 (zeitgeber time) and cross-linked with 1% formaldehyde. The ChIP experiment was performed by using the EpiQuik Plant Chromatin Kit (Epigentek, Farmingdale, NY, USA). The normal mouse lgG was used as the negative control. The mouse anti-MYC and anti-HA antibody (Santa Cruz Biotechnology, Dallas, TX, USA) were used to immunoprecipitate the immunocomplexes. The purified chromatin fragments were subjected to qPCR. The primers used in the ChIP experiments are listed in Supplementary Materials Table S1.

### 4.7. Protoplast Transfection Assays

To generate reporter and effector constructs, the 5 kb promoter sequence of *ERF1* was amplified by PCR and cloned into the pGreenII 0800-LUC vector; the corresponding CDS sequences of *PIF1*, *PIF3*, *PIF4*, *PIF5,* and *GFP* were cloned into pGreenII 62-SK vectors. Protoplast isolation from Col-0 and PEG mediated transformation was performed as described [55], and a total of 11 ug plasmid (1 ug reporter plasmid, and 10 ug of each effector plasmid) was used to perform the transformation. To measure the LUC and Renilla luciferase activities, a Dual-Luciferase Reporter Assay System (Promega, Madison, WI, USA) was used, and the luciferase activities were analyzed by GloMax-96 Microplate Luminometer (Promega, Madison, WI, USA). All the experiments were performed at least three biological times. The primers used are listed in Supplementary Materials Table S1.

## Supplementary Materials

The following are available online at https://www.mdpi.com/2223-7747/9/9/1246/s1. Figure S1. PIF5 directly binds to *ERF1* promoter. Figure S2. *HA-ERF1* can partially rescue the enhanced susceptibility of *PIF5-GFP* against Botrytis. Table S1. Primers used in this study. Data S1. The differentially expressed genes in *pifq* plants.
