# Supplementary material for: The PIFs Redundantly Control Plant Defense Response against Botrytis cinerea in Arabidopsis
Source: Plants (Basel). 2020 Sep 21;9(9):1246. doi: 10.3390/plants9091246 (PMC7570020; doi:10.3390/plants9091246)
Supplement: Supplementary file 1 [file plants-09-01246-s001.zip › plants-928749-suplementary-proofed/plants-928749-supplementary.docx]

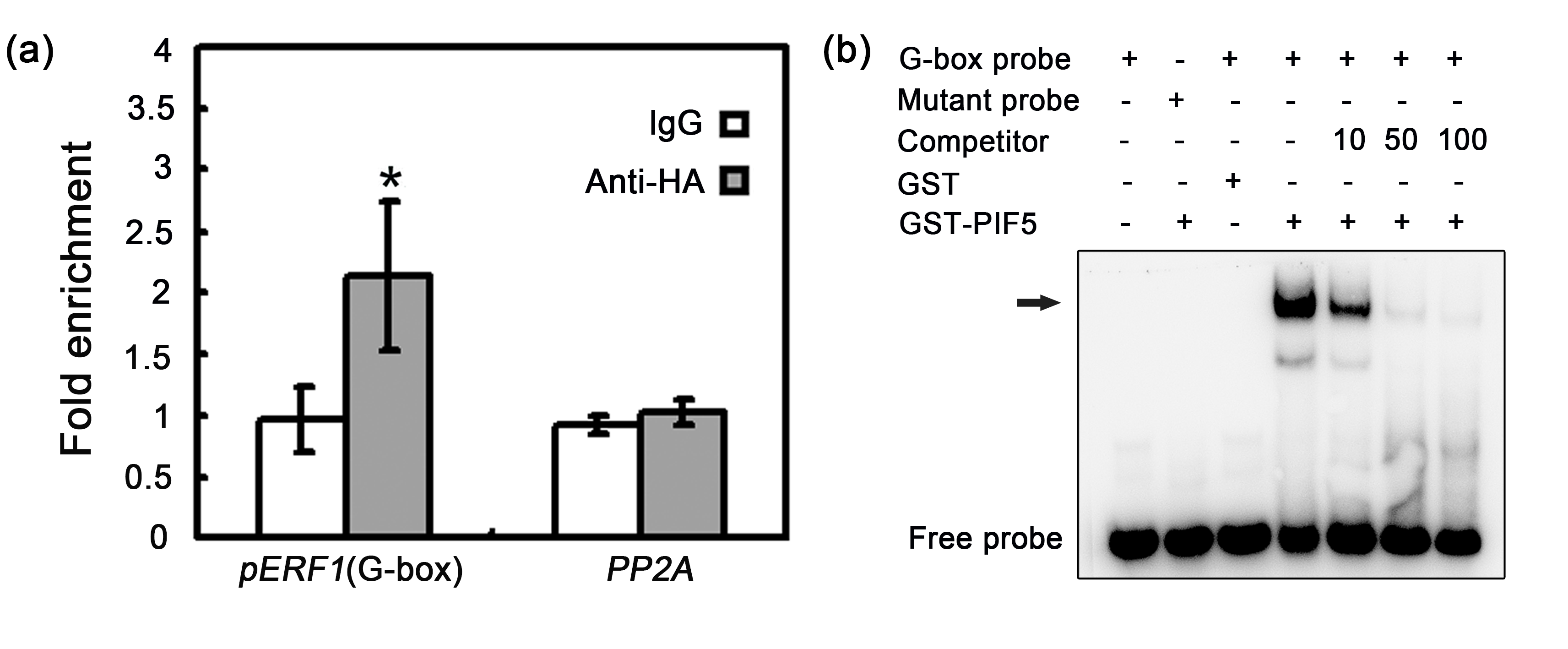


**Figure 1.** PIF5 directly binds to *ERF1* promoter. (**a**) ChIP-qPCR using *PIF5-HA* overexpression plants shows that PIF5 binds to the *ERF1* promoter region containing the G-box in vivo. The error bars indicate SD of three independent experiments. Asterisks indicate Student’s *t*-test significant differences (**p*<0.05). (**b**) EMSA assay shows that GST-PIF5 binds to the promoter of *ERF1* in vitro.


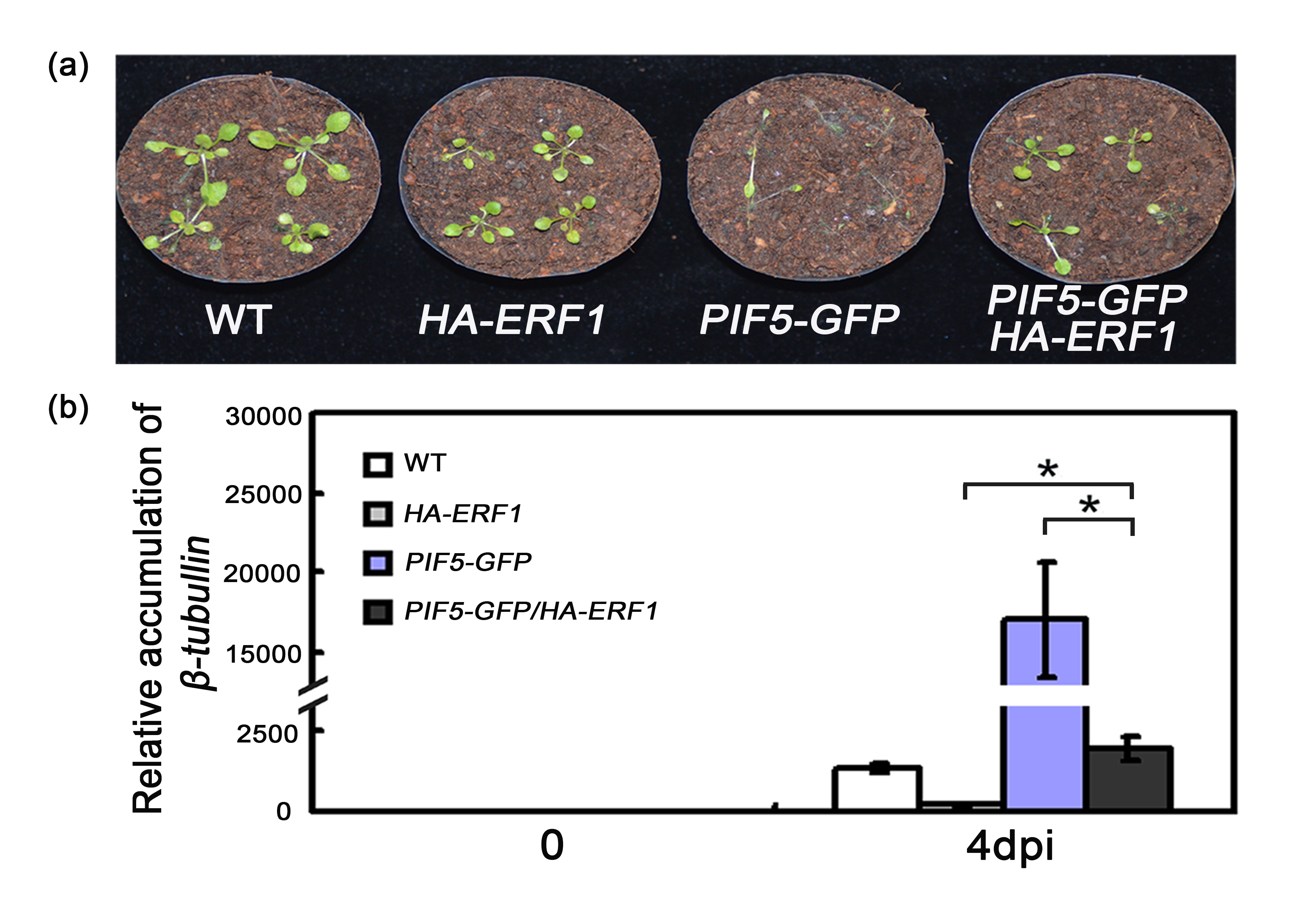


**Figure 2.** *HA-ERF1* can partially rescue the enhanced susceptibility of *PIF5-GFP* against Botrytis. (**a**) The disease symptoms of spray-inoculated plants. (**b**) The *B. cinerea β-tubulin* mRNA accumulation analysis. Error bars indicate SD of three independent experiments. Asterisks indicate Student’s *t*-test significant differences (**p* < 0.01).

**Table 1.** Primers used in this study.

| **Use** | **Gene name** | **Primers (5’->3’)** |
| --- | --- | --- |
| Transgenetic plants | *PIF1*  *PIF5* | Sac1 PIF1-G-F: AAGAGCTCATGCATCATTTTGTCCCTGACTTCGAT  Xba1 PIF1-G-R: AATCTAGAACCTGTTGTGTGGTTTCCGTGATC  Sac1 PIF5-G-F: AAGAGCTCATGGAACAAGTGTTTGCTGATTGGAATTTT  Bamh1 PIF5-G-R: AAGGATCCGCCTATTTTACCCATATGAAGACTGTCG |
| Transient expression assay | *PIF1*  *PIF3*  *PIF4*  *PIF5*  *ERF1* | Sac1 PIF1-62sk-F: AAGAGCTCATGCATCATTTTGTCCCTGACTTCGAT  Xba1 PIF1-62sk-R: AATCTAGATTAACCTGTTGTGTGGTTTCCGTG  Sac1 PIF3-62sk -F: AAGAGCTCATGCCTCTGTTTGAGCTTTTCAGG  BamHI PIF3-62sk -R: AAGGATCCTCACGACGATCCACAAAACTGATCAGAAG  Sac1 PIF4-62sk -F: AAGAGCTCATGGAACACCAAGGTTGGAGTTTTG  XbaI PIF4-62sk -R: AATCTAGATCAGTGGTCCAAACGAGAACCGTC  Sac1 PIF5-62sk-F: AAGAGCTCATGGAACAAGTGTTTGCTGATTGGAATTTT  BamHI PIF5-62sk-R: AAGGATCCTCAGCCTATTTTACCCATATGAAGACTG  0800-ERF1-LUC- Sal1-F: AGTCGACaatcccgtaagtcctacgcgt  0800-ERF1-LUC-BamHI-R: AGGATCCgtagaaaaaatactctgtttcttgactactct |
| EMSA | *ERF1* | ERF1-G-box-probe-F: GAGCCAAAAACTTTGAACACGTGCGGGATATCAAATAATCA  ERF1-G-box-probe -R: TGATTATTTGATATCCCGCACGTGTTCAAAGTTTTTGGCTC  ERF1-mutant probe-F: GAGCCAAAAACTTTGAATGACCTCGGGATATCAAATAATCA  ERF1- mutant probe-R: TGATTATTTGATATCCCGAGGTCATTCAAAGTTTTTGGCTC  ERF1-competitor-F: GAGCCAAAAACTTTGAACACGTGCGGGATATCAAATAATCA  ERF1-competitor-R: TGATTATTTGATATCCCGCACGTGTTCAAAGTTTTTGGCTC |
| CHIP | *ERF1*  *PP2A* | pERF1(G-box)-F: GAAGTTTAAAATAGAGCCAAAAACTTTG  pERF1(G-box)-R: TCTATTAGAACACTACACATCATCACATG  PP2A-F: CAACGAACAAATCACAGAAAACATG  PP2A-R: AAAGGTAAAGAAGACAGCAACGAATT |
| RT-qPCR | *β-tubulin*  *ERF1*  *ORA59*  *PDF1.2*  *HEL*  *ERF5*  *ERF6*  *ERF104*  *PIF1*  *PIF3*  *PIF4*  *PIF5*  *ACTIN2*  *IPP2* | β-tubulin-F: TCTTGAGAGCGGTGGTATC  β-tubulin-R: TTGCATACGATCGGAGATACCT  ERF1-F: TCTCTTCCCTTCAACGAGAACG  ERF1-R: GATTTGATCGGAAGGTCTTGACT  ORA59-F:AAAAGAAGAAGGAAAAGAAGCCAC  ORA59-R: GTGTCGAATGTCCCAAGCCA  PDF1.2-F: TCACCCTTATCTTCGCTGCTCT  PDF1.2-R: ATGATCCATGTTTGGCTCCTTC  HEL-F: ATAATCCGGCGCAGAATAATTG  HEL-R: CAGTTACTGCAGCATTTGTTCT  ERF5-F : GTTGGGAAGTGGAAACCACG  ERF5-R : GGAAACGTCTCTCCACCGTT  ERF6-F : CGTCGTGGAACTCGTGTTTG  ERF6-R : TTGTACAGGCCACGACCATC  ERF104-F : TTGGGACTTACGACACTGCC  ERF104-R : GGCGGAGAACCCTTATCTCG  PIF1-F: CCCCAACATGTTAGCCGCTC  PIF1-R: TTGACGGGTCAGAAGCATGA  PIF3-F: TATGCAATGGGCTTGCCTGA  PIF3-R: GCCACTGGTTGTTGTTGCAT  PIF4-F: GAGCCCGGTACAGTTACCTC  PIF4-R: GTACCGGGTTTTGGCAAACT  PIF5-F: CCCGGGGTACAATCATCTCC  PIF5-R: GGAGCGGACCGGTTCATAA  Actin2-F: TGTGCCAATCTACGAGGGTTT  Actin2-R: TTTCCCGCTCTGCTGTTGT  IPP2-F: GTATGAGTTGCTTCTCCAGCAAAG  IPP2-R: GAGGATGGCTGCAACAAGTGT |
| Identification of mutants | *pil5-2*  *pif1-1*  *pif3-7*  *pif4-2*  *pif5-3* | pil5-2-L: ATCTTTCTGGGGTTTTTCTTTTG  pil5-2-R: AAAATGCGCTGAAAACTACAAAG  Salk_KO: AAACGTCCGCAATGTGTTAT  pif1-1-L: ACTTAAAAAGGGCTTTCAAGACT  pif1-1-R: CCAGTATCTTCGACGAATGAGTC  Sail_KO: TGAATTTCATAACCAATCTCGATACAC  pif3-7 wt-F: AGAAGCAATTTGGTCACCATGCTC  pif3-7 wt-R: TGCATACAAATAGTCGATCGTATG  pif3-7 del-F: GGTGTGTATGTGAGAAGGTACATCCAT  pif3-7 del-R: AAGCTTAGCTTTGGTGAGCCTGAAAAGCTC  pif4-2-L: GAAGGAGTCGAGCAGCTGAAGTTC  pif4-2-R: AAAGTGGCTCACCAACCTAGTGG  Sail_KO: TGAATTTCATAACCAATCTCGATACAC  pif5-3-L: CCGACTGTTATAACCGAGGATCT  pif5-3-R: AACCGAGAAGGTTTTGGAGATAG  Salk_KO: AAACGTCCGCAATGTGTTAT |
